# Influence of Antibiotic Management on Microbial Selection and Infectious Complications After Trauma

**DOI:** 10.3389/fmed.2021.678382

**Published:** 2021-09-10

**Authors:** Cora Rebecca Schindler, Mathias Woschek, Jan-Niklas Franz, Philipp Störmann, Dirk Henrich, Ingo Marzi

**Affiliations:** Department of Trauma, Hand and Reconstructive Surgery, University Hospital Frankfurt, Frankfurt, Germany

**Keywords:** polytrauma, traumatic brain injury, Interleukin-6, severely injured patient, Interleukin-10, inflammation

## Abstract

**Background:** The inflammatory response and post-traumatic complications like infections play an important role in the pathophysiology of severe injuries. This study examines the microbiological aspects in anti-infective treatment of trauma patients and their inflammatory response in post-traumatic infections complications.

**Patients and Methods:** A retrospective analysis of prospectively collected data in trauma patients (ISS ≥ 16) over a 1-year period (01/2018 to 12/2018) is provided. Patient population was stratified into severely injured patients without post-traumatic infection (inf-PT), and severely injured patients who developed an infection (inf+PT).

**Results:** Of 114 trauma patients, 45 suffered from post-traumatic infection during the first 10 days of hospitalization. Severely injured patients with concomitant traumatic brain injury (PT+TBI) showed the highest rate of post-traumatic infection. Pro-inflammatory reaction was tracked by levels of Interleukin (IL-)6 (day 3: inf+T 190.8 ± 359.4 pg/dL > inf-PT 56.2 ± 57.7 pg/mL (mean ± SD); *p* = 0.008) and C-Reactive-Protein (CRP, day 3: inf+PT 15.3 mg/dL > inf-PT 6.7 mg/dL, *p* = 0.001) which were significantly higher in trauma patients who develop an infectious complication and showed a significant positive correlation with the occurrence of infection. The leading entity of infection was pneumonia followed by infections of the urinary tract mainly caused by gram-negative *Enterobacteriaceae*. 67.5% of all trauma patients received single-shot antibiosis during initial care in trauma bay. The development of secondary colonization was not relevant positively correlated with single-shot antibiosis (*r* = 0.013, *p* = 0.895) and prophylactically calculated antibiotic administration (*r* = 0.066, *p* = 0.500).

**Conclusion:** Severely injured trauma patients have an increased risk for development of infectious complications, which mainly is pneumonia followed by infection of the urinary tract mainly caused by gram-negative *Enterobacteriaceae*. Based on the data in this study, the one-time antibiotic and prophylactic calculated use of antibiotics, like Cephalosporins must be critically discussed in terms of their role in the development of post-traumatic infections and microbial selection.

## Introduction

Polytrauma (PT) and severe traumatic brain injury (TBI) caused by road traffic accidents and falls are the main causes of death and disability with immense socioeconomic impact through loss of productivity, medical and rehabilitation costs (1, 2). The treatment of patients with multiple organ injuries poses a particular challenge due to different injury patterns and severity, but also due to the complex immune response (3). Post-traumatic exaggerated immunomodulation often leads to postinjury complications, multiple organ failure (MOF) or sepsis and are predictors of mortality in trauma within the first days in Intensive Care Unit (ICU) (4). The mortality rate of septic trauma patients in hospital is still high (19.5–23%). Early prevention of sepsis development can facilitate further treatment of patients and contribute to improved treatment outcomes (5). The prevention of trauma-related infections/sepsis has so far mainly involved infection prevention (e.g., surgical management, prophylactic antibiotics, tetanus vaccination) and the prevention of organ dysfunction (e.g., drugs, temporary intravascular shunts, lung protective strategies) (6). The integrity of the intestinal barrier and the intestinal microbiome are fundamental prerequisites for a functioning immune system and the maintenance of colonization resistance to bacterial pathogens (7). Trauma, perfusion damage, or surgery favor a barrier disorder of the intestinal mucosa, and bacterial translocation and subsequent infections via the bloodstream (8). Dysbiosis and the development of multi-resistant bacteria (MRE) due to the use of antibiotics can lead to severe post-traumatic infections and complications (9).

Detecting the severity of injuries at an early stage, predicting risk factors of post-traumatic complications, and preventing secondary damageare of highest clinical interest. Serum biomarkers of inflammatory reaction are an optimal complement to detect potential complications like infection in an early stage (10–12). Systemic inflammatory reactions can be tracked by leukocytosis and release of acute phase proteins (APPs), such as c-reactive protein (CRP) and cytokines (13–15). Pro- and anti-inflammatory mediators such as Interleukin-6 and -10 (IL-6, IL-10) are released in response to tissue injury and lead to both reactive and restorative inflammatory processes (6, 16, 17).

The aim of this study was to stratify a cohort of trauma patients according to the development of post-trauma infections and to evaluate them with respect to their injury pattern, treatment with anti-infective therapy and bacterial colonization and clinical outcome. We hypothesize that peri-traumatic prophylactic or calculated antibiotic therapy influences the development of post-traumatic infections and secondary bacterial colonization. In addition, we assess the post-traumatic infections by biomarkers of post-traumatic inflammation IL-6, IL-10, CRP, and WBC.

## Patients and Methods

### Ethics

The study was performed at the University Hospital Frankfurt, Goethe University after approval by the Institutional Review Board (89/19) in accordance with the Declaration of Helsinki and following STROBE guidelines (18). Written informed consent was obtained for enrolled patients or their legally authorized representatives in accordance with ethical standards.

### Patients and Study Setting

We retrospectively reviewed a cohort of severely injured trauma patients admitted to the emergency department (ED) and level-1-trauma center of the University Hospital Frankfurt from January to December 2018. All clinical data were collected prospectively as part of the quality documentation of our in-house trauma registry. All trauma patients in the ED were treated according to the Advanced Trauma Life Support (ATLS® American College of Surgeons, Chicago, IL, USA) standard and the polytrauma guidelines (19). Injury severity from trauma was calculated using the Injury Severity Score (ISS), the New Injury Severity Score (NISS) and the Abbreviated Injury Scale (AIS) score which assigns each injury a severity level between 1 (mild) and 6 (maximum) in different regions (head, face, thorax, abdomen, extremities, and external injuries) (20–22). To calculate a mortality prognosis RISC II Score (Revised Injury Severity Classification, Version 2) was used (23).

We evaluated all patients with a history of acute trauma with an ISS ≥ 16 admitted to the emergency department during this 12-month period. Participating patients had to be ≥18 years of age. Patients who met this inclusion criteria were stratified into 2 cohorts: severely injured patients without post-traumatic infection (inf-PT), and severely injured patients who developed a relevant post-traumatic infection during their in-hospital stay (inf+PT). Relevant infections included respiratory tract infection/pneumonia (Examination material: sputum, tracheal secretion, bronchial lavage), urinary tract infection, soft tissue infection (Examination material: swab, biopsy), catheter-associated infection (Examination material: catheter tip; except urinary catheters), and bacteremia (Examination material: blood culture). In addition, infections were designated as “unknown” if patients had clear symptoms of infection such as fever or laboratory signs of inflammation of unclear origin. Severe open fracture was defined by fractures with an AIS ≥2 and a soft tissue injury of type 2 or greater according to the Gustilo-Anderson classification. Recent major surgery describes a major impact by surgical intervention performed early (within 5 days) after trauma, such as osteosyntheses of long bones, spine, or pelvis, hemi or bi-craniotomies or major intra-abdominal or -thoracal interventions. These are intended to represent a possible second hit. We defined relevant post-traumatic disability at the time of hospital discharge. Retrospectively, we evaluated several indicators to assess patient's outcome. These included the Barthel index ≤90% as a measure of limitations in self-care and activities of daily living, the Glasgow outcome scale extended (GOSE ≤4) for patients with TBI. As well as any impairment in physical function that led to discharge to inpatient rehabilitation, nursing home, or prescription of home care.

Patients with known pre-existing immunological disorders, immunosuppressive medication, burns, concomitant acute myocardial infarction, or thromboembolic events were excluded.

### Blood Processing, Serum Marker, and Microbiological Analysis

Serial venous blood samples were obtained from traumatized patients on admittance to the ED for day 1–10 after trauma. Following baseline sample, subsequent blood was collected following the standard hospital procedures in pre-chilled tubes (silicate coated granules and polyacrylester gel; S-Monovette©, Sarstedt Inc., Nümbrecht, NRW, Germany) and stored on ice. Blood was centrifuged at 2,000 × g for 15 min at 4°C and the supernatant (serum) was stored at −80°C until analysis. Frozen serum samples were not thawed for this study before analysis. Interleukin (IL-)6 and Interleukin (IL-)10 concentrations were measured by IL-6/IL-10 Eli-pair Enzyme-linked Immunosorbent Assay (ELISA) (Diaclone, Hoelzel Diagnostica, Cologne, Germany) according to the manufacturer's instructions.

Levels of C-Reactive Protein (CRP, reference range <0.5 mg/mL), IL-6 (reference range <7 pg/mL), and blood count (leucocytes/white blood cells (WBC), reference range 3.96–10.41/nL) are part of routine diagnostics and were collected retrospectively from patients' medical records. Serial blood analyses for these parameters of clinical routine were not available for each of these patients. Microbiological diagnostics like bronchial lavage (BAL), urinary, and blood culture test were ordered only for patients in which potentially serious infection was suspected. Nasal, pharyngeal, and rectal swabs (Multidrug resistant gram-negative bacteria (MRGN) Screening) routinely taken in our clinic upon admission via the trauma ward, to the intensive care unit and then weekly. Tests were performed by chemical pathology and microbiology laboratories adjacent to the hospital where the trauma center is located. Results (incl. data regarding antibiotic therapy) were extracted retrospectively in chart review. A positive microbial result was defined pathogenic bacterium could be cultured in the laboratory. In classifying a bacterium as pathogenic, we considered the symptoms of the patient as recorded in the patient's medical records, the presence of other potential pathogens from other microbiological tests for the same patient, the frequency with which the bacterial species was isolated, the epidemiology associated with the specific bacterium. In cases where the source of the infection could not be identified, it was recorded as “unknown.”

### Statistical Analysis

Continuous normally distributed variables were summarized using means ± standard deviation (SD), while categorical or continuous variables with skewed distributions were summarized using medians with interquartile ranges (IQR). The *p*-values for categorical variables were derived from the two-sided Fisher's exact test, and for continuous variables from the Mann–Whitney *U*-test or the Kruskal–Wallis test in case of more than two group comparisons. Significant values were adjusted by the Bonferroni *post-hoc* test. Spearman's rank correlation coefficients were calculated to determine correlations between biomarkers and clinical characteristics. Receiver–operator characteristic (ROC) curves were generated to analyze the variables predictive power for the development of post-traumatic infection. *p* < 0.05 was considered to be statistically significant (^*^ = p <0.05; ^**^ = p <0.01; ^***^ = p <0.001). Values are reported as mean for continuous variables and as percentages for categorical variables. All analyses were performed using the Statistical Package for Social Sciences (SPSS for Mac©), version 26 (SPSS Inc., Chicago, IL).

## Results

### Demographics and Clinical Injury Characteristics

Table 1 shows the demographic and clinical characteristics stratified by the incidence of post-traumatic infection in trauma patients.

**Table 1:** Demographic and injury characteristics stratified by the incidence of post-traumatic infection.

	**inf-PT** (***n*** **= 69)**	**inf+PT** (***n*** **= 45)**	
	***% of n***	***% of n***	***p*** **-value**
Sex (m)	72.5%	80.0%	0.355
Penetrating injury	7.6%	6.7%	0.069
Severe open fracture/Decollement	18.6%	25.0%	0.429
Open TBI	31.4%	20.5%	0.190
Recent major surgery	47.8%	77.8%	<0.001
**Outcome**			
In-hospital Mortality	33.3%	2.2%	<0.001
Severe Disability	5.7%	43.2%	<0.001
	**Median (IQR)**	**Median (IQR)**	***p*** **-value**
Age (y)	49 (22–73)	47 (30–69)	0.586
ISS (pts)	24 (17–32)	27 (22–34)	0.044
NISS (pts)	29 (22–41)	34 (27–43)	0.315
AIS_head_ (pts)	4 (2-4)	3 (0–5)	0.889
AIS_thorax_ (pts)	2 (0–4)	2 (0–3)	0.952
AIS_abdomen_ (pts)	0 (0–0)	0 (0–2)	0.035
AIS_extremities_ (pts)	2 (0–2)	2 (0–3)	0.053
RISC II (%)	4.5 (0.6–67.5)	5.5 (1.0–14.2)	0.916
ETI (d)	0 (0–2)	5 (1–5)	<0.001
ICU (d)	2 (0–6)	14 (7–20)	<0.001
_syst_ RR_ER_ (mmHg)	139 (115–165)	127 (106–160)	0.128
Puls_ER_ (bpm)	85 (75–100)	88 (66–116)	0.752
Blood transfusion (250ml bag)	2.5 (2–5)	0 (0–2)	0.005

*M, male, IQR, Interquartile Range; y, years; pts, points; TBI, traumatic brain injury; “recent major surgery”: Major surgery on head, long bones, spine, pelvis, thorax, or abdomen within 5 days after trauma; AIS, Abbreviated Injury Scale; ISS, Injury Severity Score; RISC II, Revised Injury Severity Classification, Version 2; ICU, Intensive Care Unit; ETI, Endotracheal Intubation; d, days; _syst_RR, systolic blood pressure; bpm, beats per minute; ER, at admission to Emergency room*.

During the 12-month study period, *n* = 114 severely injured patients (ISS ≥ 16) met the inclusion criteria. Of these, *n* = 45 patients developed any post-traumatic infection (inf+PT cohort) during the first 10 days of hospitalization. For *n* = 69 patients no relevant infection or microbiological findings were documented (inf-PT cohort).

The majority of these trauma patients (inf-PT 72.5% and inf+PT 80.0%) were male. The included patients most suffered blunt injury trauma, whereas the incidence of penetrating trauma (e.g., gunshot or stab wounds) was <8% in both groups. No relevant difference in the incidence of open fractures or major soft tissue injuries (inf-PT 18.6% and inf+PT 25.0%, *p* = 0.429) or TBIs (inf-PT 31.4% and inf+PT 20.5%, *p* = 0.190) could be shown between the two groups. Significant more patients with post-traumatic infections (77.8% > 47.8%, *p* < 0.001) underwent major surgery during their in-hospital stay. No significant difference in median (IQR) age [inf-PT 49 (22–73) years vs. inf+PT 47 (30–69) years; *p* = 0.586] was seen. Median (IQR) ISS of inf-PT cohort was slightly lower [inf-PT: ISS 24 (17–32) pts. < inf+PT: ISS 27 (22–34) pts., *p* = 0.044] but no significant difference was found in NISS (*p* = 0.315). Median (IQR) risk of mortality (RISC II) was <6% (*p* = 0.916) for patients in both groups. The median (IQR) length of stay in intensive care unit (inf-PT 2 (0–6) days < inf+PT 14 (7–20) days, *p* < 0.001) and ventilation time (inf-PT 0 (0–2) < inf+PT 5 (1–5), *p* < 0.001) was significantly extended in inf+PT cohort. There was no relevant difference in indicators of hemorrhagic shock, such as blood hemoglobin fraction (*p* = 0.565), systolic blood pressure (RR_syst_, *p* = 0.128) and pulse (*p* = 0.752). Patients without post-traumatic infections received significant more blood transfusions [median (IQR) 2.5 (2–5) > 0 (0–2), *p* = 0.005].

### Higher Level of Inflammation Markers in Trauma Patients With Posttraumatic Infection

**Figure 1:**
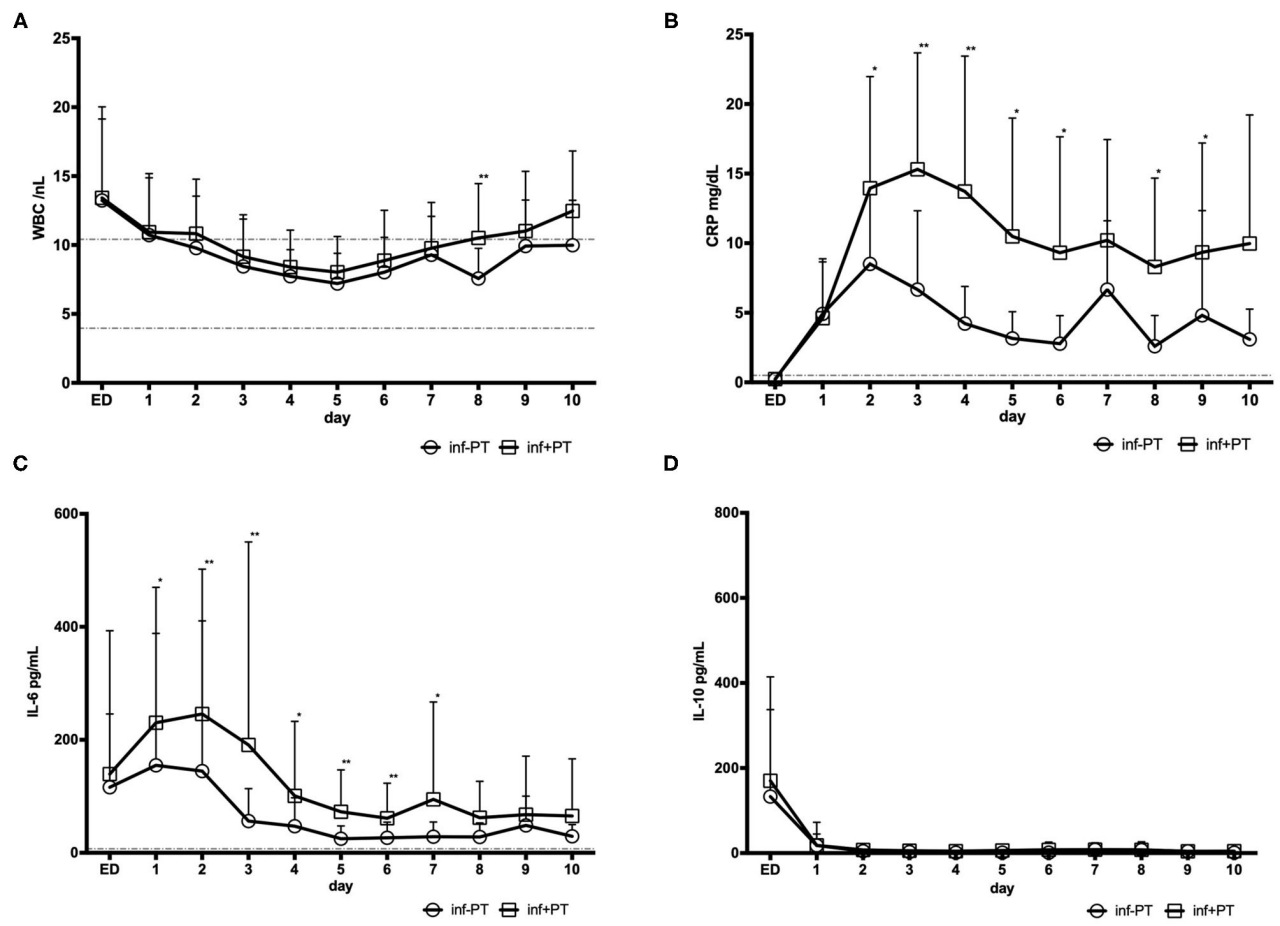
Inflammation markers in trauma patients with (inf+PT) and without (inf-PT) post-traumatic infection **(A)** Leukocytes with reference range 3.96–10.41/ nL **(B)** CRP (C-Reactive Protein) in mg/dL with reference range <0.5 mg/dL **(C)** Interleukine(IL-)6 with reference range <7 pg/mL and **(D)** Interleukine(IL-)10 (no reference range).

Stratification of the cohorts was supported and post-traumatic infection was tracked by serum biomarkers of pro- and anti-inflammatory response. Figure 1 shows the expression profile of he markers in trauma patients with (inf+PT) compared to patients without (inf-PT) post-traumatic infection.

Blood count analyses showed that the kinetic profile of the WBCs showed to relevant difference betweenboth cohorts (except day 8, which will most likely be an outlier). CRP level in patients with post-traumatic infection supports the positive microbial findings in this cohort and was significantly higher (day 3: inf+PT 15.3 mg/dL > inf-PT 6.7 mg/dL, *p* = 0.001) comparing trauma patients without infection. Spearman's rank correlation showed significant positive correlation between CRP level and the occurrence of post-traumatic infection from day 2–6, 8, and 9.

As well the IL-6 level was significantly higher in inf+PT group (day 3: inf+PT 190.8 ± 359.4 pg/dL > inf-PT 56.2 ± 57.7 pg/mL, *p* = 0.008). A significant positive correlation between pro-inflammatory IL-6 level and the occurrence of post-traumatic infection from day 1 to 7 (day 3: *r* = 0.384; *p* = 0.007) was shown.

Anti-inflammatory IL-10 levels didn't show significant difference in both study cohorts.

### Post-traumatic Infections in Trauma Patients

Table 2 shows the entities and number (*n*) of post-traumatic infection in trauma patients stratified by injury pattern (PT, PT+TBI, isTBI). Table 3 shows the spectrum of microorganisms found in the cultures of bronchial lavage, tracheal secretion, urine, intraoperative wound swab, catheter, and blood of trauma patients with post-traumatic infection (inf+PT, *n* = 45).

**Table 2:** Post–traumatic infections in trauma patients stratified by injury pattern.

	**All**		**PT**		**PT+TBI**		**isTBI**	
**Entity**	***n***	**% of** ***N***	***n***	**% of** ***N***	***n***	**% of** ***N***	***n***	**% of** ***N***
Infections	45	39.1	16	44.4	24	51.1	5	16.1
Pneumonia	28	19.4	8	22.2	17	36.2	3	9.7
Urinary tract	10	8.8	2	5.5	6	12.8	2	6.5
Soft tissue	7	6.1	7	19.4	0		0	
Intravenous catheter	3	2.6	0		3	6.4	0	
Unknown	5	4.4	1	2.8	3	6.4	1	3.2
Bacteremia	5	4.4	0		5	10.6	0	
Multiple organ infections	9	6.3	2	5.5	6	12.8	1	3.2

*All (N = 114) trauma patients with ISS ≥ 16 stratified by injury pattern: PT (N = 36) = multiple injured patients (polytrauma) without TBI, PT+TBI (N = 47) = multiple injured patients with TBI, isTBI (N = 31) = isolated TBI*.

**Table 3:** Spectrum of microorganisms in trauma patients with post-traumatic infections (*N* = 45).

**Microorganisms**	***n***	**% of** ***N***	***n*** **(%) Pneum**	***n*** **(%) Urin**	***n*** **(%) Tiss**	***n*** **(%) Cat**	***n*** **(%) Bac**
**Gram-negative bacteria**							
*Enterobacteriaceae*	28	62.2	14 (50.0)	9 (90)	3 (42.9)	1 (33.3)	1 (20.0)
*Enterobacter aerogenes*	3	6.7	1 (3.6)	1 (10)		1 (33.3)	1 (20.0)
*Enterobacteriaceae ssp*.	6	13.3	4 (14.3)	2 (20)			
*Enterococcus faecium (VRE)*	2	4.4		1 (10)	1 (14.3)		
*Escherichia coli (ESBL/3MRGN)*	8	17.8	4 (14.3)	3 (30)	1 (14.3)		
*Klebsiella pneumoniae*	6	13.3	3 (10.7)	1(10)	1 (14.3)		
*Klebsiella ssp*.	3	6.7	2 (7.1)	1 (10)			
*Haemophilus influenzae*	1	2.2	1 (3.6)				
*Moraxella catarrhalis*	1	2.2	1 (3.6)				
*Proteus mirabilis*	1	2.2		1 (10)			
*Pseudomonas aeruginosa*	4	8.9	3 (10.7)		1 (14.3)		
**Gram-positive bacteria**							
*Bacillus cereus*	1	2.2			1 (14.3)		
*Corynebacterium tuberc*.	1	2.2			1 (14.3)		
*Propionibacterium acnes*	2	4.4			1 (14.3)		1 (20.0)
*Staphylococcaceae*	8	17.8					
*Staphylococcus aureus*	3	6.7	3 (10.7)				
*Staphylococcus epidermidis*	3	6.7				2 (66.7)	3 (60.0)
*Staphylococcus ssp*.	2	4.4					2 (40.0)
*Streptococcus pneumoniae*	1	2.2	1 (3.6)				
Polymicrobial coinfections	14	31.1	8 (28.6)	3 (30)	2 (28.6)		1 (20.0)

*Pneum, Pneumonia; Urin, Infection of urinary tract; Tiss, soft tissue infection; Cat, Catheter-associated infection; Bac, Bacteremia; ESBL, Extended Spectrum ß-Lactamase; 3MRGN, Multi-Resistant Gram-Negative bacteria; VRE, Vancomycin-Resistant Enterococcus*.

*N* = 45 of 114 patients suffered from post-traumatic infection during the first 10 days after trauma. Among these, the trauma patients with concomitant traumatic brain injury (PT+TBI) had the highest rate (44.4%) of post-traumatic infections. The leading entity of post-traumatic infection was pneumonia (PT+TBI 36.2% > PT 22.2% > TBI 9.7%), which was followed by infections of the urinary tract (PT+TBI 12.8%, TBI 6.5%) and soft tissue (PT 19.4%).

The most common pathogens detected were gram-negative *Enterobacteriaceae* (62.2%), like *E. coli* (17.8%) and *Klebsiellae* (32.1%). 50% of pneumonia and 90% of urinary tract infections were caused by this gram-negative *Enterobacteriaceae*. 8.9% of inf+PT suffered from an infection with *Pseudomonas aeruginosa*. The microbial spectrum of soft tissue infections was spread over both gram-negative and gram-positive bacteria. Among the gram-positive bacteria mainly *Staphylococcaceae* (17.8%), such as *Staphylococcus aureus and St. epidermidis* were found. A proven bacteremia was only found in PT cohort (*n* = 5), which was most likely catheter-associated (*n* = 3), caused by gram-positive *Staphylococcus* and gram-negative *Enterobacteriaceae*. 6.3% of inf+PT suffered from multiple organ infections (PT+TBI 12.8% > PT 5.5.% > isTBI 3.2%). In 31.1% of inf+PT a polymicrobial coinfection with several pathogenic bacteria was found, including those affecting several organ systems.

### Indications and Spectrum of Anti-infective Therapy in Trauma Patients

Table 4 shows antibiotic therapy in trauma patients with and without post-traumatic infections. Table 5 shows the antibiotic spectrum in therapy of peri-traumatic anti-infective management.

**Table 4:** Indications of antibiotic therapy in trauma patients.

	**All**		**inf-PT**		**inf+PT**	
**Indication**	***n***	**% of** ***N***	***n***	**% of** ***N***	***n***	**% of** ***N***
Single shot (ER)	77	67.5	45	65.2	32	71.1
Prophylactic/Calculated	63	55.3	32	46.4	31	68.9
Perioperative	5	4.4	4	5.8	1	2.2
Proven infection	38	33.3	0		38	84.4
Multiple antibiotic treatment	25	21.9	2	2.9	23	51.1

*All (N = 114) trauma patients ISS ≥ 16; ER, Emergency room; BLI, Beta Lactamase Inhibitor; PT, Polytrauma patients; infPT, Polytrauma patients with post-traumatic infection*.

**Table 5:** Antibiotic spectrum of anti-infective management in trauma patients.

	**All** (***N*** **= 114)**		**inf-PT** (***N*** **= 69)**		**inf+PT** (***N*** **= 45)**	
**Substance**	***n***	**% of** ***N***	***n***	**% of** ***N***	***n***	**% of** ***N***
*Cephalosporins*	45	39.5	20	29.0	25	55.6
*1. Generation*	3	2.6	1	1.4	2	4.4
*2. Generation*	38	33.3	19	27.5	19	42.2
*3. Generation*	4	3.5	0		4	8.9
*Nitromidazole (Metronidazol)*	12	10.5	4	5.8	8	17.8
*Carbapenems*	45	39.5	28	40.6	17	37.8
*Meropenem*	40	35.1	27	39.1	13	28.9
*Imipenem*	5	4.4	1	1.4	4	8.9
*Penicillin + BLI*	25	21.9	4	5.8	21	44.4
*Lincosamide (Clindamycin)*	3	2.6	0		3	6.7
*Fluoroquinolone*	13	11.4	0		13	28.9
*Ciprofloxacin*	7	6.1	0		7	15.6
*Levofloxacin*	6	5.3	0		6	13.3
*Glycopetide antibiotics*	10	8.8	0		10	22.2
*Vancomycin*	3	2.6	0		3	6.7
*Teicoplanin*	7	6.1	0		7	15.6
*Cotrimoxazol*	4	3.5	0		4	8.9
*Ansamycine (Rifampicin)*	1	0.9	0		1	2.2
*Antibiotics of last resort*	3	2.6	0		3	6.7

*BLI, Beta Lactamase Inhibitor; inf-PT, Polytrauma patients without post-traumatic infections; inf+PT, Polytrauma patients with post-traumatic infection*.

67.5% of all 114 trauma patients received single-shot antibiosis during initial care in trauma bay. *Cefuroxime* (inf-PT 26.1% and inf+PT 42.2%) and *Meropenem* (inf-PT 34.8% and inf+PT 26.6%) were the most commonly used antibiotics. Stratified by injury pattern, patients without TBI were mainly medicated with *Cefuroxime* (63.9%) while patients with TBI received predominantly *Meropenem* (58.1%). In case of open fracture or extensive soft tissue damage, *Metronidazole* was given additionally (4.8%).

In 55.3% (inf-PT 30.4% and inf+PT 17.9%) the anti-infective therapy was continued prophylactically/calculated to avoid later infection-associated complications or based on the clinical picture of an impending infection. In addition to the above-mentioned indications for the calculated application of *Meropenem* (inf-PT 31.9% and inf+PT 11.1%) and *Cefuroxime* (inf-PT 5.8% and inf+PT 15.5%), *Penicillin* + *BLI* (inf-PT 5.8% and inf+PT 42.2%) was frequently used. 4.4% of all trauma patients received peri-operative antibiosis. In 33.3% the antibiotic treatment was associated with a proven infection by positive bacterial culture. Drug spectrum is broader in the case of a confirmed infection and, due to the mostly present infection with gram negative anaerobes (shown in Table 2), mainly dominated by *Fluoroquinolones* and *Glycopeptide antibiotics*.

### Bacterial Carriage in Trauma Patients

Table 6 shows the results of routine nasal, pharyngeal and rectal swabs taken on admission to our trauma bay and during the stay in the intensive care unit.

**Table 6:** Bacterial carriage in trauma patients (*N* = 114).

**Characteristics**	***n***	**% of** ***N***
Primary colonization	28	24.6
Secondary colonization	8	7.0
**Microorganisms**		
*Escherichia coli (ESBL/3MRGN)*	15	13.2
*Enterococcus faecium/faecalis (VRE)*	7	6.1
*Pseudomonas aeruginosa*	3	2.6
*Acinetobacter baumannii*	2	1.8
*Citrobacter freundii*	1	0.9
*Enterobacter cloacae*	1	0.9
*Staphylococcus aureus (MRSA)*	1	0.9
*Proteus mirabilis*	1	0.9

*ESBL, Extended Spectrum ß-Lactamase; 3MRGN, Multi-Resistant Gram-Negative bacteria; VRE, c; MRSA, Methicillin-Resistant Staphylococcus Aureus*.

In *n* = 28 of 114 trauma patients a primary bacteria colonization was found, mainly with *Escherichia coli* (*E. coli*; *ESBL* = Extended Spectrum ß-Lactamase; *3MRG*N = Multi-Resistant Gram-Negative bacteria), followed by *Enterococcus faecium/faecalis* (*VRE* = Enterococcus faecium/faecalis) and *Pseudomonas aeruginosa* in the rectal smear. In *n* = 8 trauma patients secondary colonization occurred during hospitalization. The detection of colonization by other bacteria, like *Acinetobacter baumannii, Citrobacter freundii, Enterobacter cloacae* and *Proteus mirabilis* was <2%. MRSA (Methicillin-Resistant Staphylococcus aureus) was detected in only *n* = 1 patient. Of these 28 patients with a positive smear, 17 developed a post-traumatic infection.

Of the *n* = 8 patients with secondary colonization, *n* = 6 patients received single-shot antibiotics in trauma bay, *n* = 2 patients received prophylactically calculated antibiotics (because of an open wound). All of them developed a post-traumatic infection during first 10 day after trauma. A statistically significant positive correlation (Spearman's rank correlation, *r* = 0.251; *p* = 0.007) could be shown between a positive screening test result and the occurrence of post-traumatic infection. The development of secondary colonization was not relevant positively correlated with single-shot antibiosis (*r* = 0.013, *p* = 0.895) and prophylactically calculated antibiotic administration (*r* = 0.066, *p* = 0.500).

## Discussion

### Risk Factors for Posttraumatic Infections

Of the trauma patients included in this study, the majority were male and under 50 years of age; one-third suffered a post-traumatic infection. Polytrauma is the leading cause of death in Western countries for people up to 45 years of age, in a male-to-female ratio of 2.6:1 (1). Percentage of mortality was significantly higher in the inf-PT group. This initially rather surprising result is most likely explained by the fact that a high percentage of severely injured patients in the inf-PT group died in the early stages after trauma. Thus, they do not suffer post-traumatic infections and bias the mortality rate. One could even assume that the already high infection rate could be even higher. Patients who have extended hospital stay with prolonged periods of coma, immobilization and ventilation are at enormous risk for post-traumatic complications, like pulmonary infections (24). On the other hand, it is quite plausible that patients who have suffered a post-traumatic infection also have a higher disability rate. Longer periods of respiratory weaning and comorbidities often complicate rehabilitation, including critical illness myopathy, which leads to Intensive care unit acquired weakness (ICUAW) (25). Open fractures and major skin injuries do not appear to have a relevant influence on the development of an infectious post-traumatic complication. One reason for this could be the consistent, calculated antibiotic therapy (Cefuroxime & Metronidazole) applied from the outset for this type of injury. In this study, the impact of a major surgical intervention in a relevant temporal context of 5 days after trauma was investigated. Hereby, all patients were included who were treated according to a dynamic therapy evaluation in the sense of “safe-definitive-surgery.” We showed that in patients with post-traumatic infections, surgical interventions were performed significantly more frequently in close temporal relation to the trauma. Early total care (ETC) involves definitive surgical stabilization of all long bone fractures in the early phase of treatment (24–48 h). Damage control surgery (DCS) refers to those maneuvers designed to ensure the survival of the (bleeding) patient with vital-threatening organ injury or long bone and pelvic fractures. The advantage of ETC seemed to be fewer pulmonary complications, shorter duration of ICU, and hospital stay (26). Advances in understanding the pathophysiologic and immunologic mechanisms after severe injury led to a shift from ETC to DCS. Traumatic injury leads to systemic inflammatory response syndrome (SIRS), followed by a recovery phase mediated by a counter-regulatory anti-inflammatory response (CARS). Severe inflammation can lead to acute organ failure and early death after injury. A mild inflammatory response followed by excessive CARS can produce a long-lasting, deleterious immunosuppressed state and promote the development of infections. The initial trauma is referred to as the “first hit” and predisposes the patient to a potential risk of deterioration (27). Major post-trauma interventions in the form of a “second-hit” phenomenon, i.e., due to fat emboli and hypoxic events, can lead to serious complications such as pulmonary dysfunction, coagulopathy, fat or pulmonary embolism, excessive immune reactions and post-traumatic infections. Over the last three decades, extensive research has been published on the “second hit” phenomenon, the pathophysiology of trauma and the consequences of subsequent interventions (28–30). The safe-definitive-surgery (SDS) concept was presented as a dynamic combination of the ETC and DCO care strategies. Regular reevaluation and assessment of the patient's physiology and condition can combine the advantages of the DCO and ETC procedures, which could allow “safe definitive care” (SDS) of the severely injured patient.

### Early Tracking of Posttraumatic Infection by Biomarkers Is Efficient

We further analyzed the validity of the routine laboratory and recognized biomarkers of post-traumatic immune response for their value in these patients with post-traumatic infections. For the critically ill trauma patient who survives for more than 3 days, infectious complications are the leading cause of death after serious head injury (31). The standard clinical diagnosis of pneumonia includes fever, leukocytosis, purulent secretions, and new or progressive infiltrates in a chest x-ray (32). Nevertheless, biomarkers are certainly the optimal complement to clinical and radiological findings to assess the course and outcome of the injury and its complications sufficiently (10, 33). We could show that levels of IL-6 and CRP are significantly higher in trauma patients who develop post-traumatic infection. Both markers showed a significant positive correlation with the presence of post-traumatic infection. This result supports our stratification of the patient cohort. Furthermore, the results show that already on day 1 and 2 a differentiation of the markers between PT+Inf and PT-Inf occurs, which in these cases suggests an early enhanced immune response in the PT+Inf group. It was shown before that primary trauma injury triggers an immunomodulatory response that is associated with an increase in CRP (34). Interleukins play a particularly prominent role in the development of post-traumatic complications und and have been widely studied in relation to trauma (15, 16, 35). In the acute phase reaction, IL-6 is known to regulate inflammation and immunity. IL-6 is secreted in increased amounts early after trauma (36, 37). Previous studies described a correlation of IL-6 with the severity of injury (ISS) and mortality, especially during the first post-traumatic hours (15, 36, 38, 39). Additionally, it has potentially predictive value for post-traumatic infection (40, 41). Our results underline the importance of these biomarkers, which can already be early indicators of a complication, while clinical parameters may still be compensated, and microbiological results are pending. However, the early increase of inflammatory markers in PT+Inf basically questions the efficacy of the calculated single shot antibiosis, which was broadly applicated in both groups. Furthermore, the application of calculated prophylactic antibiotic therapy, which was consistently given in the TBI group, which most frequently suffered from pneumonia, remains questionable.

IL-10 is commonly known as an anti-inflammatory cytokine that exerts immunomodulatory functions and is particularly important in the resorption phase (42, 43). However, IL-10 does not appear to have any diagnostic value for the development of post-traumatic infection in this study. WBC analysis is one of the most basic biomarker tracking of post-traumatic complications. Studies showed that leukocytosis can predict the severity of injury in trauma victims (44). On the other hand, Paladino et al. showed that WBC counts are not useful in the diagnostic of trauma, as patients with severe injuries had significantly higher WBC level than those with minor injuries, but both were in the normal range (45). In our study, we saw that after trauma, WBCs are only slightly above normal (especially in the first 24 h). Furthermore, the WBC level of inf+PT does not differ significantly from that of inf-PT and therefore has less diagnostic value in the assessment of a post-traumatic infection.

### Post-traumatic Infections Are Mainly Caused by Gram-Negative Enterobacteriaceae

The leading cause of post-traumatic infection was pneumonia mainly caused by gram-negative Enterobacteriaceae. Stratified by injury pattern severely injured patients with concomitant traumatic brain injury (PT+TBI) shows the highest rate of post-traumatic infection. These findings support Helling et al., who analyzed infectious complications in the severely head injured and found that pulmonary infections occur most commonly, affecting 41% of the patients. The predominantly offending organism was gram-negative bacteria (46). In addition, the urinary tract is also a common source and must always be considered as a relevant differential diagnosis. The trauma patient has an increased risk of infection and the most common cause of late death after trauma is sepsis. Transfusion, hypotension and prolonged ventilatory support are predictive of septic complications. In addition, open wounds, additional surgical procedures, extensive invasion through various tubes and drains, post-traumatic immune modulation, transfusions, medication, and suboptimal nutritional status contribute to an increased risk of infection. Victims of blunt trauma have a significantly higher risk of post-traumatic infection than patients with penetrating trauma (24). Our results do not support the currently discussed idea, that blood transfusions facilitate the development of infections (47). In contrast, it could be concluded that the longer time of endotracheal intubation (ETI) and ICU stay of inf+PT are logically related to the complications of the infection. Longer ventilation times lead to an increased risk of infection after trauma and thus to a longer stay in the intensive care unit (32). However, the higher rate of mortality in the PT group inevitably leads to shorter ICU treatment.

### Prophylactic Antibiotic Treatment vs. Microbiome Selection in Trauma Care

The prevention of post-traumatic complications such as infections remains one of the most important challenges in primary as well as intensive care. In the literature, the prophylactic use of antibiotics does not appear to change the development of infections in trauma patients and in many cases, antibiotic therapy is empirical, depending on the patient's condition (31). In our study most patients received prophylactic anti-infective therapy with Cefuroxime. The benefit of a perioperative antibiotic prophylaxis of Cefuroxime has already been proven and is already established in guidelines in Europe (48). The excitation spectrum can vary from place to place. In our institution the most common pathogens are gram-negative Enterobacteriaceae, just like already published in the study by Helling et al. (46). Staphylococcus and other gram-positive bacteria were found to be the main cause of wound infections in inf+PT cohort. Therefore, antibiotics covering the gram-positive and gram-negative bacterial spectrum should be administered as early as possible in infection prophylaxis. However, this does not only affect patients with open injuries. According to our study, however, the general prophylactic administration in all severely injured patients with massive injuries and the risk of reperfusion damage or second hits should also be discussed. A second-generation Cephalosporine is good practice basis. In heavily contaminated wounds this can be supplemented with an Aminoglycoside. If an anaerobic infection is suspected, a combination with e.g., Metronidazole is considerable (49). In our study, patients with TBI mainly received Meropenem. Typical pathogens of acute bacterial meningitis in open traumatic brain injury (TBI) are Staph. aureus, coagulase-negative Staphylococci, aerobic gram-negative bacteria which respond mainly to Meropenem and Cephalosporins (50).

Meropenem is important in the treatment of post-neurosurgical meningitis and penetrates the cerebrospinal fluid (CSF) (51). However, there is currently no evidence-based guideline for the prophylactic administration of antibiotics in trauma patients with or without TBI. Fever without not due to infectious events is common in trauma patients. Reasons for this could be an excessive immune reaction, as described above. In this study we found a high rate of patients receiving calculated Penicillin + beta lactamase inhibitor (BLI). Penicillin is a broad-spectrum antibiotic and often given in connection with unclear infections to cover a possible broad spectrum of pathogens. In the group of inf+PT, prophylactic, and calculated prophylactic antibiotics were administered relatively more frequently, i.e., in the case of already existing risk factors such as open fractures, large wounds or open TBI. Whether or not a single-shot antibiotic was administered did not initially affect the incidence for the occurrence of post-trauma infection. In addition, single-shot antibiotics and prophylactically calculated antibiotic application were not relevant positively correlated with the development of secondary colonization. However, in this study, it appears that prophylactic administration, especially of Cephalosporins, results in selection pressure in favor of gram-negative Enterobacteriaceae.

An important pathophysiological factor in the development of post-traumatic complications is the dysfunction of the external (skin) and internal paracellular blood and organ barriers, including the blood, air, and intestinal blood barriers in the brain (BBB), leading to the flooding of the tissue with immune cells and microbial invasion (52). IL-6 modulates the expression of tight junction proteins and the release of adhesion molecules in the plasma of polytrauma patients (ISS ≥ 18) correlates with disease severity and organ dysfunction (53). In addition, hemorrhagic shock is associated with intestinal barrier dysfunction and the development of MODS after trauma (53). Additional selection pressure favoring pathogenic and multiresistant bacteria further increases the risk of serious infectious complications.

Based on the data in this study, the one-time antibiotic and prophylactic calculated use of antibiotics, like Cephalosporins must be critically discussed in terms of their role in the development of post-traumatic infections by microbial selection (1, 2, 4, 10, 14–17, 31–36, 38–41, 51, 52, 54, 55). The treatment of patients with multiple organ injuries poses a particular challenged (45).

### Limitations of the Study

The most important limitation is the retrospective nature of the data analysis; however, all clinical data were acquired in a prospective manner, and only the cytokines were measured later-on. The variance of the individual values is large, specifically of markers, which is due to potential confounders such as volume administration, blood products, shock, which are difficult to standardize. In addition, the values for CRP and WBC are part of routine diagnostics and were collected retrospectively from the patients' medical records. As a result, a complete number *n* = 114 could not be collected for each day over time, which may have resulted in higher standard deviations with lower significance. The sample of this single center study came from an ICU facility specializing in traumatology. This may have a limiting influence on the generalizability of our study results, and it is possible that these results are not applicable to all trauma situations.

## Conclusion

Severely injured trauma patients have an increased risk for development of infectious complications. Severely injured patients with concomitant traumatic brain injury (PT+TBI) show the highest rate of post-traumatic infection. In this context, for the first time, we showed that broad application of single-shot antibiotics and prophylactic calculated antibiotic use need to be critically discussed in terms of their role in the development of post-traumatic infections and microbial selection for gram negative anaerobic bacteria. The leading entity of infection was pneumonia followed by infection of the urinary tract and soft tissue infections, mainly caused by gram-negative Enterobacteriaceae. Levels of IL-6 and CRP are significantly higher in trauma patients who develop an infectious complication. Both markers showed the efficacy of early biomarker tracking in the prediction of post-traumatic complication like infektons.

## Data Availability Statement

The original contributions presented in the study are included in the article/supplementary material, further inquiries can be directed to the corresponding author/s.

## Ethics Statement

The studies involving human participants were reviewed and approved by Institutional Review Board, University Hospital Frankfurt, Goethe University (89/19). The patients/participants provided their written informed consent to participate in this study.

## Author Contributions

IM, CS, and DH designed the study, obtained the ethical approval for human analyses, established the methods, and revised the manuscript. CS collected samples, carried out data analyses, performed the statistical analysis, and made the first draft of the manuscript. CR and MW carried out experiments. PS, DH, and IM critically reviewed the manuscript. MW, PS, and J-NF contributed intellectually to the completion of the study. All authors contributed to the article and approved the submitted version.

## Conflict of Interest

The authors declare that the research was conducted in the absence of any commercial or financial relationships that could be construed as a potential conflict of interest.

## Publisher's Note

All claims expressed in this article are solely those of the authors and do not necessarily represent those of their affiliated organizations, or those of the publisher, the editors and the reviewers. Any product that may be evaluated in this article, or claim that may be made by its manufacturer, is not guaranteed or endorsed by the publisher.
